# The Acidophilic Methanotroph *Methylacidimicrobium tartarophylax* 4AC Grows as Autotroph on H_2_ Under Microoxic Conditions

**DOI:** 10.3389/fmicb.2019.02352

**Published:** 2019-10-18

**Authors:** Sepehr S. Mohammadi, Rob A. Schmitz, Arjan Pol, Tom Berben, Mike S. M. Jetten, Huub J. M. Op den Camp

**Affiliations:** Department of Microbiology, Institute for Water and Wetland Research, Radboud University, Nijmegen, Netherlands

**Keywords:** *Methylacidimicrobium*, methanotrophic Verrucomicrobia, acidophilic, H_2_, [NiFe] hydrogenases, oxygen sensitivity

## Abstract

Emissions of the strong greenhouse gas methane (CH_4_) to the atmosphere are mitigated by methanotrophic microorganisms. Methanotrophs found in extremely acidic geothermal systems belong to the phylum Verrucomicrobia. Thermophilic verrucomicrobial methanotrophs from the genus *Methylacidiphilum* can grow autotrophically on hydrogen gas (H_2_), but it is unknown whether this also holds for their mesophilic counterparts from the genus *Methylacidimicrobium*. To determine this, we examined H_2_ consumption and CO_2_ fixation by the mesophilic verrucomicrobial methanotroph *Methylacidimicrobium tartarophylax* 4AC. We found that strain 4AC grows autotrophically on H_2_ with a maximum growth rate of 0.0048 h^–1^ and a yield of 2.1 g dry weight⋅mol H_2_^–1^, which is about 12 and 41% compared to the growth rate and yield on methane, respectively. The genome of strain 4AC only encodes for an oxygen-sensitive group 1b [NiFe] hydrogenase and H_2_ is respired only when oxygen concentrations are below 40 μM. Phylogenetic analysis and genomic comparison of methanotrophs revealed diverse [NiFe] hydrogenases, presumably with varying oxygen sensitivity and affinity for H_2_, which could drive niche differentiation. Our results show that both thermophilic and mesophilic verrucomicrobial methanotrophs can grow as autotrophs on H_2_ as a sole energy source. Our results suggest that verrucomicrobial methanotrophs are particularly well-equipped to thrive in hostile volcanic ecosystems, since they can consume H_2_ as additional energy source.

## Introduction

Atmospheric concentrations of the strong greenhouse gas methane (CH_4_) are increasing due to anthropogenic activities (Kirschke et al., 2013). With a changing climate, it is essential to understand alterations in the sources and sinks of the global methane cycle (Murrell and Jetten, 2009). Methanogenic Archaea constitute the largest methane source, producing this odorless gas in a vast variety of habitats, such as wetlands, rice fields, oceans and the digestive system of termites and ruminants (Conrad, 2009). The main sink can be found in the troposphere, where methane is oxidized photochemically by hydroxyl radicals (OH) (Voulgarakis et al., 2013). In addition, methanotrophs significantly mitigate methane emissions to the atmosphere by oxidizing methane produced by methanogens (Murrell and Jetten, 2009). Methanotrophs either oxidize methane with oxygen (Hanson and Hanson, 1996; Op den Camp et al., 2009; Semrau et al., 2010) or anaerobically, utilizing a variety of alternative electron acceptors (Knittel and Boetius, 2009; Ettwig et al., 2010; Haroon et al., 2013). For many years it was believed that methane oxidation was a feature restricted to the subphyla Alpha- and Gammaproteobacteria (Op den Camp et al., 2009). However, this view was rejected due to the description of novel aerobic methanotrophs belonging to the phylum Verrucomicrobia (Dunfield et al., 2007; Pol et al., 2007; Islam et al., 2008). All known verrucomicrobial methanotrophs of the genus *Methylacidiphilum* (strains Kam1, SolV, V4 and sp. RTK17.1) have been isolated from extremely acidic geothermal ecosystems (Pol et al., 2007; Op den Camp et al., 2009; Sharp et al., 2013; Carere et al., 2017). Methanotrophy by thermophilic members of the Verrucomicrobia phylum is an extreme affair, since they can grow on methane below pH 1 and at temperatures up to 65°C (Op den Camp et al., 2009).

All verrucomicrobial methanotrophs of the genus *Methylacidiphilum* were isolated from hot and acidic environments. Recently, 16S rRNA sequences were retrieved from acidic geothermal areas with a moderate temperature, revealing closely related verrucomicrobial methanotrophs (Sharp et al., 2012, 2014). Strain LP2A was isolated from such a moderate-temperature area and indeed only grows at a moderate temperature (Sharp et al., 2014). This novel verrucomicrobial methanotroph shares only 89.6% 16S rRNA gene sequence identity with representatives of the thermophilic genus *Methylacidiphilum* (Sharp et al., 2014). Shortly after this finding, van Teeseling et al. (2014) isolated and characterized three new species of acidophilic verrucomicrobial methanotrophs from diverse soil patches at the Solfatara crater, located at the center of the Campi Flegrei caldera, near Naples (Italy). Interestingly, these novel verrucomicrobial methanotrophs are unable to grow at high temperature and they are therefore all mesophiles. The 16S rRNA genes of the new isolates are only about 89% similar to those of the *Methylacidiphilum* species and 97–98% identical to that of strain LP2A. Therefore, the new genus name *Methylacidimicrobium* was proposed and the four mesophilic strains were described as *Methylacidimicrobium tartarophylax* 4AC, *Methylacidimicrobium fagopyrum* 3C, *Methylacidimicrobium cyclopophantes* 3B and *Methylacidimicrobium* strain LP2A (van Teeseling et al., 2014). Maximum growth rates (μ_max_) of 0.013–0.040 h^–1^ were observed at temperature optima between 35 and 44°C, respectively (van Teeseling et al., 2014). Strain 4AC is the most acid-tolerant methanotroph known to date, growing at pH values as low as 0.5. Similar to the thermophilic verrucomicrobial strains SolV and V4, all isolated mesophilic strains grow autotrophically using the Calvin-Benson-Bassham cycle for CO_2_ fixation (Khadem et al., 2011; Sharp et al., 2012; van Teeseling et al., 2014). Interestingly, these novel isolates all encode for hydrogen-oxidizing enzymes.

In many volcanic habitats, H_2_ is available as an additional energy source for methanotrophs (Chiodini et al., 2001; Hanczár et al., 2002; Carere et al., 2017). Hydrogenases catalyze the oxidation of H_2_ to two protons plus two electrons, and vice versa. Genomic analyses have revealed a plethora of hydrogenases in many different phyla, with either [NiFe], [FeFe] or [Fe] as metals in the active site (Lubitz et al., 2014; Greening et al., 2016). Several methane oxidizers are able to consume H_2_, as was demonstrated for *Methylosinus* sp. (de Bont, 1976), *Methylocystis* sp. and *Methylococcus capsulatus* Bath (Csáki et al., 2001; Kelly et al., 2005). The presence of genes encoding an uptake hydrogenase and the ribulose-1,5-bisphosphate carboxylase (RuBisCO) in several proteobacterial methanotrophs indicates the possibility of autotrophic growth. However, whereas autotrophic growth of *M. capsulatus* Bath was observed on solid agar media, physiological studies in liquid media did not support this observation (Dalton and Whittenbury, 1976; Taylor et al., 1981; Stanley and Dalton, 1982; Baxter et al., 2002). The thermophilic verrucomicrobial strains SolV and RTK17.1 were shown to grow autotrophically on H_2_ (Carere et al., 2017; Mohammadi et al., 2017). Moreover, these extremophiles can utilize CH_4_ and H_2_ simultaneously. Reducing equivalents derived from the oxidation of H_2_ could aid in growth of methanotrophs in times when CH_4_ availability is low (Hanczár et al., 2002). In geothermal environments with large fluctuations in H_2_ and CH_4_ emissions, this mixotrophic lifestyle can provide a major advantage over less metabolically versatile microorganisms (Ward et al., 2017; Power et al., 2018). Consequently, the competence of oxidizing both CH_4_ and H_2_ could give an explanation for the dominance of verrucomicrobial methanotrophs in terrestrial volcanic ecosystems (Semrau et al., 2011; Carere et al., 2017).

The closed genome of the thermophilic strain SolV encodes for two uptake hydrogenases that catalyze H_2_ oxidation: an oxygen-tolerant group 1d and an oxygen-insensitive group 1h H_2_-uptake [NiFe] hydrogenase (Anvar et al., 2014; Mohammadi et al., 2017). The draft genomes of the mesophilic verrucomicrobial methanotrophs (strains 4AC, 3B, 3C and sp. LP2A) also revealed the presence of an H_2_-uptake hydrogenase, but it differs significantly from the group 1d and group 1h [NiFe] hydrogenases of the thermophilic strains (Sharp et al., 2014; van Teeseling et al., 2014). Interestingly, the mesophilic strain 4AC was shown to be highly sensitive to O_2_, whereas the thermophilic strain SolV was shown to consume H_2_ at ambient air (van Teeseling et al., 2014; Mohammadi et al., 2017). The presence of an uptake hydrogenase and a carbon fixation pathway in all mesophilic strains suggests that they can grow as autotrophs on hydrogen gas. This suggestion implies that hydrogen oxidation by mesophilic verrucomicrobial methanotrophs can be important in the mitigation of greenhouse gas emissions from the natural geothermal environment. We therefore hypothesized that the mesophilic methanotroph *Methylacidimicrobium tartarophylax* 4AC can grow as an autotroph on H_2_ as sole energy source under oxygen-limited conditions. To investigate this hypothesis, various physiological experiments were performed in continuous and batch cultures, in the presence or absence of H_2_, CH_4_ and CO_2_ at different oxygen concentrations.

## Materials and Methods

### Microorganism and Medium Composition

*Methylacidimicrobium tartarophylax* strain 4AC used in this study was initially isolated and enriched from diverse soil spots at the Solfatara crater, which is at the center of the Campi Flegrei caldera, near Naples (Italy) (Pol et al., 2007; van Teeseling et al., 2014). In this study, the medium was composed of 0.2 mM MgCl_2_ ⋅ 6 H_2_O; 0.2 mM CaCl_2_ ⋅ 2 H_2_O; 1 mM Na_2_SO_4_; 2 mM K_2_SO_4_; 2 mM (NH_4_)_2_SO_4_ (10 mM to reach OD_600_ of 5) and 1 mM NaH_2_PO_4_ ⋅ H_2_O. A trace elements solution was used containing 1 μM NiCl_2_, CoCl_2_, Na_2_MoO_4_, ZnSO_4_ and CeCl_3_, 5 μM MnCl_2_ and FeSO_4_, 10 μM CuSO_4_ and 50 μM nitrilotriacetic acid (NTA). The pH of the medium was adjusted to 3.0 using 1 M H_2_SO_4_. To avoid precipitation, CaCl_2_ ⋅ 2 H_2_O and the rest of medium were autoclaved separately and mixed after cooling. This medium composition contained all nutrients to obtain an OD_600_ of 1.0 and was used in batch and continuous cultures, unless stated otherwise.

### Chemostat Cultivation on Methane and Hydrogen Gas

The reactor system (Applikon Biotechnology, Delft, NL) was operated at 38°C with a stirring speed of 470 rpm. During growth on methane, the oxygen-limited continuous culture (liquid volume of 950 ml) was supplied with medium at a flow rate of 15.6 ml ⋅ h^–1^ (*D* = 0.016 h^–1^). A gas supply of 6% CH_4_ (v/v), 5% CO_2_ (v/v) and 3% O_2_ was provided by mass flow controllers (MFCs) through a sterile filter and sparged into the medium at a gas flow rate of approximately 13 ml ⋅ min^–1^. A dissolved oxygen concentration (dO_2_) of approximately 0–0.02% was obtained when the cells reached a steady state. During growth on hydrogen, the oxygen-limited chemostat (liquid volume of 1.3 L) was supplied with medium at a flow rate of 5 ml ⋅ h^–1^ (*D* = 0.004 h^–1^). A gas supply of 15% H_2_ (v/v), 5% CO_2_ (v/v) and 3% O_2_ was provided by MFCs through a sterile filter and sparged into the medium at a gas flow rate of 13.2 ml ⋅ min^–1^. A dO_2_ of about 0–0.01% was obtained when cells reached a steady state. The cell-containing medium was removed automatically from the chemostat by a peristaltic pump when the liquid level reached the sensor in the reactor. During growth on methane or hydrogen gas, the pH was regulated in the steady state at 2.8 and 4.0 using 0.2 M NaOH, respectively.

### Batch Cultivation

To start a continuous culture on hydrogen gas, we initially tried to grow strain 4AC in batch mode in the bioreactor used for the chemostat cultivation. To obtain the maximum growth rate (μ_max_) on methane, cells were grown at 38°C at 470 rpm without any limitation in the medium using a gas supply in which the O_2_ concentration was below 5%. In order to obtain the μ_max_ on hydrogen, cells were grown at 38°C at 470 rpm without any limitation in the medium while the dO_2_ was kept at 0–0.01%. The batch growth was repeated at least two times for four generations.

### Gas Analysis

The consumption of hydrogen gas and carbon dioxide was measured using a HP 5890 gas chromatograph (Agilent, Santa Clara, United States) equipped with a Porapak Q column (1.8 m, ID 2 mm) and a thermal conductivity detector. For hydrogen gas analysis, 30–40 μl gas samples were injected with a glass syringe. The consumption of methane was analyzed using a HP 5890 gas chromatograph (Agilent, Santa Clara, United States) equipped with a Porapak Q column (1.8 m, ID 2 mm) and a flame ionization detector. For methane analysis, 100 μl gas samples were injected with a glass syringe. The consumption of oxygen was measured on an Agilent series 6890 gas chromatograph (GC) equipped with Porapak Q and Molecular Sieve columns and a thermal conductivity detector as described before (Ettwig et al., 2008). For oxygen analysis, 50 μl gas samples were injected with a glass syringe.

### Dry Weight Determination and Elemental Analysis

To determine the biomass dry weight concentration, 10 ml of the culture suspension (triplicate) was filtered through pre-weighed 0.45 μm filters and dried to constant weight in a vacuum oven at 60°C. To measure the total content of carbon and nitrogen, 10 ml of the culture suspension (duplicate) was centrifuged at 4,500 × *g* for 30 min and the clear supernatant was used for the analysis. The nitrogen and carbon content in the supernatant was compared with the corresponding values in the whole cell suspension. The total carbon and nitrogen contents were measured using TOC-L and TNM-1 analyzers (Shimadzu, Kyoto, Japan).

### Respiration Experiments

Respiration rates were measured polarographically in a respiration cell with an oxygen microsensor (RC350, Strathkelvin, Motherwell, United Kingdom) using 3 ml of whole cell suspensions of strain 4AC (OD_600_ = 0.3). Methane-, hydrogen- or oxygen-saturated medium was injected into the respiration chamber to obtain the desired dissolved gas concentrations. The O_2_ signal was monitored and recorded using SensorTrace Basic software (Unisense, Aarhus, Denmark). The temperature and stirring rate in the respiration chamber were adjusted to 38°C and 1000 rpm, respectively. Rates were expressed as nmol O_2_ ⋅ min^–1^ ⋅ mg DW^–1^ and, when necessary, corrected for endogenous respiration. To avoid high oxygen concentrations at the start of an experiment, samples taken from cultures were immediately transferred into rubber septum sealed bottles under an anoxic atmosphere of nitrogen and carbon dioxide. These bottles contained medium in case dilution was necessary. A subsample was taken from the bottle by a syringe with a long needle and introduced into the respiration chamber at the bottom with the oxygen probe in place while pushing out the air via the inlet channel.

### Hydrogenase Classification and Phylogenetic Analysis

The NCBI accession numbers and corresponding microbial species for all [NiFe] hydrogenase large subunit sequences were retrieved from HydDB (Søndergaard et al., 2016). The accession list was initially filtered for methanotrophs by querying NCBI for species that possess both a methane monooxygenase and a methanol dehydrogenase. The resulting list contained a number of methylotrophs (with an annotated ammonia/methane monooxygenase) which were removed by manual inspection. The remaining sequences were retrieved using NCBI Batch Entrez; the sequences from verrucomicrobial methanotrophs were added manually. All sequences were aligned using the default algorithm in T-Coffee v12.00.7fb08c2 (stand-alone). After manual inspection of the multiple sequence alignment, a maximum-likelihood tree was calculated by RAxML v8.2.10. The group 3d [NiFe] hydrogenase was used as outgroup and pruned from the final tree. MEGA 7 was used to visualize the tree and collapse branches that did not contain verrucomicrobial sequences.

## Results

### *M. tartarophylax* Strain 4AC Consumes Hydrogen at Low Oxygen Conditions

**FIGURE 1:**
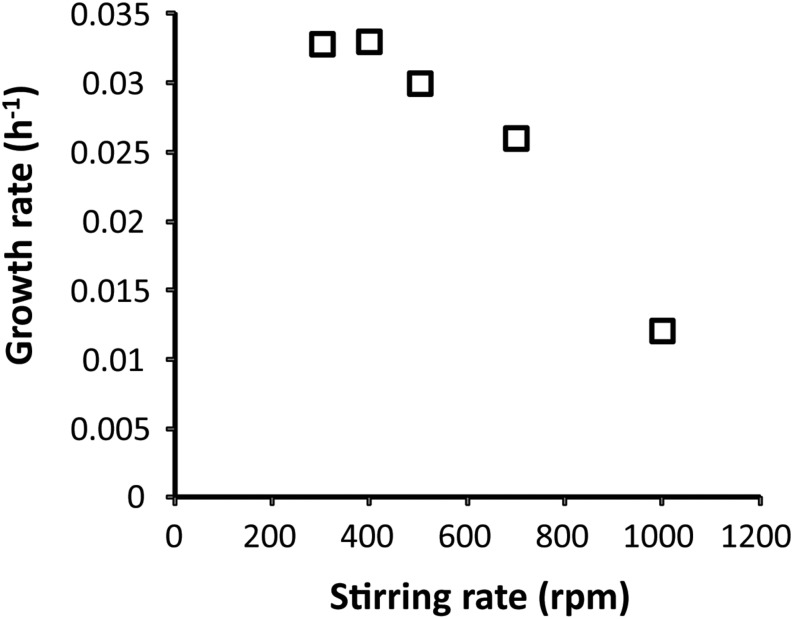
Effect of increased stirring on the growth rate of *Methylacidimicrobium tartarophylax* 4AC with methane as a substrate. Each data point represents the average of two independent experiments at the same stirring speeds.

To show H_2_ consumption under oxygen-limited conditions by strain 4AC, cells were initially cultivated on methane in the bioreactor using batch and chemostat conditions. Considering the high sensitivity to oxygen of strain 4AC (van Teeseling et al., 2014), the maximal stirring rate that could be used during the growth was determined. High stirring rates may cause oxygen stress to strain 4AC by changing the actual oxygen gradient. Lower stirring rates may allow for an oxygen gradient close to the outside of the cell. We observed that increases of the stirring rate from 400 to 1000 rpm resulted in a decrease of the growth rate on methane from 0.033 to 0.012 h^–1^, respectively (Figure 1). At stirring rates lower than 400 rpm, no further increase in the growth rate was observed compared to the μ_max_ reported previously (0.035 h^–1^, van Teeseling et al., 2014). Therefore, growth experiments were performed at 400 to 500 rpm. A comparison of batch growth on methane and hydrogen is given in Supplementary Figure S1. Cells from batch cultures of strain 4AC growing on methane at a growth rate of 0.033 h^–1^ and an oxygen concentration of 1% did not show any hydrogen oxidation when the oxygen concentration in the respiration chamber was 175 μM (Table 1). However, when the O_2_ concentration became lower than 40 μM, oxidation of 4.1 nmol O_2_ ⋅ min^–1^ ⋅ mg DW^–1^ was measurable, which is approximately 4% of the oxygen consumption with methane (105.9 nmol ⋅ min^–1^ ⋅ mg DW^–1^; Table 1). Moreover, cells from a continuous culture grown on methane under O_2_ limitation showed hydrogen respiration rates below 1 nmol O_2_ ⋅ min^–1^ ⋅ mg DW^–1^ when the oxygen concentration in the respiration chamber was above 100 μM (Table 1) and again elevated rates were measured when O_2_ concentrations were below 30 μM (4.1 and 2.8 nmol O_2_ ⋅ min^–1^ ⋅ mg DW^–1^; Table 1). Based on our observations, we assume that strain 4AC could oxidize hydrogen only when the oxygen concentration in the respiration chamber was less than 40 μM. To test this hypothesis, we transferred the methane-grown cells directly from the continuous culture under oxygen limitation to a closed serum bottle with a headspace gas of hydrogen and nitrogen. A subsample was taken from this bottle by a syringe with a long needle and introduced at the bottom of the respiration chamber. Using this procedure, we obtained a 10 μM O_2_ concentration in the respiration chamber at the start of the experiment and this clearly resulted in higher hydrogen respiration rates (8.1 nmol O_2_ ⋅ min^–1^ ⋅ mg DW^–1^; Table 1). These results strongly indicate that the hydrogenase responsible for the hydrogen oxidation activity is only active under oxygen-limited conditions.

**TABLE 1:** Oxygen respiration rates of *Methylacidimicrobium tartarophylax* 4AC cells with CH_4_ or H_2_.

**Growth condition**	**e^–^ donor**	**Limitation**	**Growth rate (h^–1^)^a^**	**dO_2_ (%)^b^**	**O_2_ (μM)^c^**	**Respiration rate^*d*^**	
						
						**H_2_**	**CH_4_**
Batch	CH_4_	None	0.033 ± 0.003	1	175 <40	≈ 0 4.1	105.9
Continuous^e^	CH_4_	O_2_	0.016	<0.02	120 <30	<1 4.1	112.9
Continuous^f^	CH_4_	O_2_	0.016	<0.02	175 <30	<1 2.8	100.6
Continuous^g^	CH_4_	O_2_	0.016	<0.02	100 <40	<1 3.9	115.7
Continuous^h^	CH_4_	O_2_	0.016	<0.02	<10	8.1	n.d.
Batch	H_2_	None	0.0048 ± 0.0006	0.05–0.1	<10	11.2	n.d.
Continuous	H_2_	O_2_	0.004	<0.02	<10	10.5	n.d.

*^*a*^In batch and continuous cultures, growth rate refers to μ and D, respectively. The concentrations of the gasses provided by mass flow controllers in the chemostat cultures were (v/v): 5% CO_2_ and 6% CH_4_ or 15% H_2_. ^*b*^For the batch culture on methane the actual oxygen concentration was regulated at 1%. For the hydrogen batch culture regulation was at 0.05–0.1%, while the hydrogen and methane continuous cultures under oxygen limitation showed values below detection limit (0.02%). ^*c*^The μM units refer to O_2_ concentrations in the liquid phase in the respiration chamber. ^*d*^The respiration rates measured in the respiration chamber are expressed in nmol O_2_ ⋅ min^–1^ ⋅ mg DW^–1^ and are averages of duplicate measurements that did not deviate more than 10%. ^*e*^Cells were aerated for 10 min prior to the respiration test. ^*f*^Cells were aerated for 1 h prior to the respiration test. ^*g*^Cells without aeration prior to the respiration test. ^*h*^Cells were transferred to a capped serum bottle (60 ml) containing an anoxic atmosphere of hydrogen and nitrogen. n.d. = not determined.*

### *M. tartarophylax* 4AC Grows Autotrophically on Hydrogen

We next asked whether strain 4AC is able to grow as an autotroph on H_2_. To answer this question, H_2_ was introduced into a bioreactor batch culture on CH_4_ with a growth rate of 0.02 h^–1^. Oxygen was supplied to the reactor so that due to consumption the dissolved oxygen value (dO_2_) was kept below the detection limit (reading value between 0 and 0.01%). The total gas inflow was kept at 13.2 ml ⋅ min^–1^. After a period of 4 days in which both H_2_ and CH_4_ were simultaneously consumed, methane was removed from the gas mixture while keeping the total gas inflow constant at 13.2 ml ⋅ min^–1^. In the batch growth of strain 4AC with H_2_ only, we measured a growth rate of 0.0048 h^–1^ (±0.0005), indicating a doubling time of 144 h. To obtain higher growth rates, we gradually increased the O_2_ supply using an MFC until dO_2_ spikes (maximum 0.02%) were being observed. At this point we stopped increasing the O_2_ supply, and after a few hours the same procedure was repeated. The optical density (OD_600_) and the consumption rates of gases were measured daily. The highest hydrogen consumption rate (using a GC) during batch growth was measured at 63 nmol H_2_ ⋅ min^–1^ ⋅ mg DW^–1^. After successful batch growth on H_2_ only, the system was switched to a continuous culture mode with a growth rate (D) at 0.004 h^–1^, which is about 83% of the μ_max_ (Table 1). During the steady state, H_2_, CO_2_ and O_2_ consumption rates were measured, resulting in rates of 36.6, 6.4 and 14.1 nmol ⋅ min^–1^ ⋅ mg DW^–1^, respectively. In both batch and continuous cultures using H_2_, the dO_2_ value was kept at zero. To confirm the high sensitivity of the hydrogenase of strain 4AC to O_2_ that was observed previously in the respiration experiments, growth in the batch condition was monitored when O_2_ was in excess. As soon as the cells were exposed to a surplus amount of O_2_, first a slow increase of the dO_2_ signal to 0.05–0.1% was observed, followed by a rapid increase showing that growth ceased due to inhibition of the hydrogenase. These results confirm the high sensitivity of the hydrogenase of strain 4AC toward oxygen and the ability of strain 4AC to grow on H_2_ as an autotroph.

### Yield

In order to quantify growth of strain 4AC on hydrogen compared to methane, the growth yields on both gasses were determined. The growth yield on CH_4_ was obtained by measuring the methane consumption of an oxygen-limited continuous culture (*D* = 0.016 h^–1^). Based on dry weight (DW) measurements a yield value of 5.1 ± 0.2 g DW ⋅ mol CH_4_^–1^ was calculated. Oxygen consumption measurements (for CH_4_ versus O_2_) and gas chromatographic analysis (for CH_4_ versus CO_2_) measurements were used to quantify CH_4_ oxidation, leading to the following stoichiometry:

$${\mathrm{CH}}_{4}+{1.55O}_{2}\to {0.66\mathrm{CO}}_{2}+{1.44H}_{2}O+{0.34\mathrm{CH}}_{2}O\left(\mathrm{biomass}\right)$$

Organic carbon analysis of centrifuged culture samples revealed the presence of 12% of the total organic matter in the supernatant. In addition, a continuous culture on H_2_ under O_2_ limitation (*D* = 0.004 h^–1^) was used to assess the growth yield on H_2_. Based on the consumption of hydrogen and dry weight measurements a yield value of 2.1 ± 0.2 g DW ⋅ mol H_2_^–1^ was calculated. Oxygen consumption measurements (for H_2_ versus O_2_) and gas chromatographic analysis (for H_2_ versus CO_2_) measurements were used to quantify H_2_ oxidation, leading to the following stoichiometry:

$$H_{2}+{0.38O}_{2}+0.17{CO}_{2}\to 0.83H_{2}O+0.17{CH}_{2}O\left(biomass\right)$$

### Hydrogenase Classification and Oxygen Sensitivity

**FIGURE 2:**
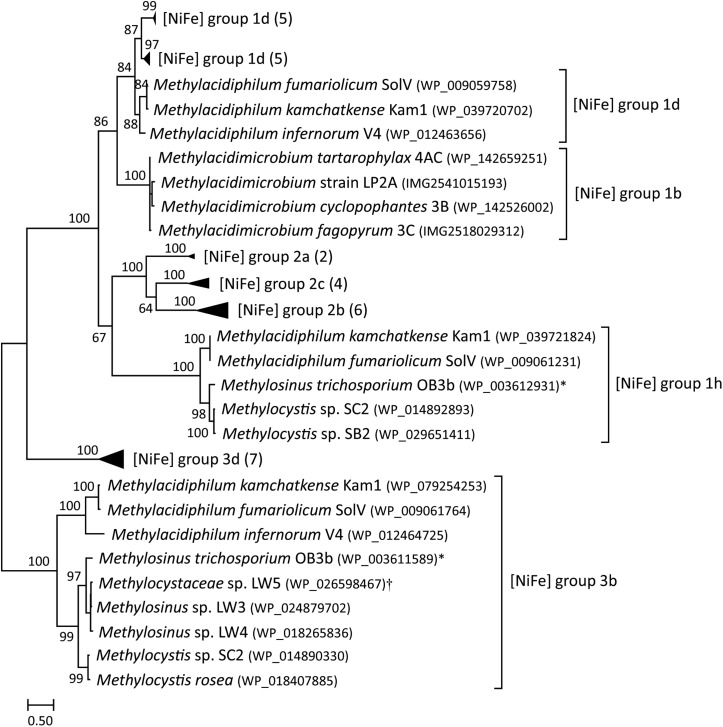
A maximum-likelihood phylogenetic tree based on the protein sequences of [NiFe] hydrogenases (large subunit) found in methanotrophs. Hydrogenase group labels are based on HydDB. The number of sequences in collapsed branches is shown in parentheses. Bootstrap scores are based on 500 replicates. ^∗^Multispecies record that also includes *Methylosinus* sp. 3S-1; ^†^Multispecies record that also includes *Methylocystis* sp. sav-2.

All known *Methylacidiphilum* isolates encode for a group 1d [NiFe] hydrogenase, whereas, all known *Methylacidimicrobium* isolates encode for a group 1b [NiFe] hydrogenase (Figure 2) (Hou et al., 2008; Anvar et al., 2014; Sharp et al., 2014; van Teeseling et al., 2014; Erikstad and Birkeland, 2015). *Methylacidiphilum* strains also encode for a group 3b [NiFe] hydrogenase that could be involved in CO_2_ fixation (Carere et al., 2017). Moreover, *M. fumariolicum* SolV and *M. kamchatkense* encode for a group 1h [NiFe] hydrogenase, proposed to be an oxygen-insensitive enzyme with a high affinity for H_2_ (Constant et al., 2010). In addition, a few other methanotrophs, such as *Methylocystis* and *Methylosinus*, possess multiple different [NiFe] hydrogenases (Figure 2). Genome analysis of the *Methylacidimicrobium* isolates revealed a membrane-bound *b*-type cytochrome protein (hupZ) in the operon of group 1b and group 1d [NiFe] hydrogenases that shuttles electrons to the terminal oxidase. The group 1h type is involved in energy conservation, but how electrons are shuttled to the electron transport chain is currently unknown (Greening et al., 2015).

Oxygen tolerance during hydrogen consumption heavily depends on the type of hydrogenase encoded (Table 2). The group 1b type studied here is active only below 40 μM O_2_, indicating that the enzyme is sensitive to oxygen or expressed only under low oxygen conditions. In contrast, the group 1h [NiFe] hydrogenase of *M. fumariolicum* SolV is active up to at least ambient oxygen. The group 1d type is classified as an oxygen-tolerant [NiFe] hydrogenase (Greening et al., 2016), but it is only expressed under oxygen-limiting conditions (Khadem et al., 2012; Mohammadi et al., 2017). Group 1d [NiFe] hydrogenases can be inactivated by O_2_, but have mechanisms to rapidly reactivate. In contrast, oxygen-insensitive hydrogenases do not get inhibited even at high concentrations (Kalms et al., 2018). The group 1b and group 1d type share clear similarities in gene arrangements and maturation proteins (Mohammadi et al., 2017). However, the large subunit of the group 1b [NiFe] hydrogenase in mesophilic strains 3B, 3C, 4AC and LP2A is only 46–48% similar to the large subunit of group 1d [NiFe] hydrogenase present in thermophilic verrucomicrobial methanotrophs. Likewise, the small subunit of the hydrogenase in strains 3B, 3C, 4AC and LP2A is only 41–44% similar to the small subunit of group 1d [NiFe] hydrogenase of *Methylacidiphilum* species. Considering the phylogenetic position, motif analysis, the lack of supernumerary iron-sulfur clusters involved in oxygen tolerance and the physiological results showing hydrogen consumption under oxygen limitation, we therefore conclude that the hydrogenases present in the *Methylacidimicrobium* strains are part of the oxygen-sensitive group 1b [NiFe] hydrogenases (Figure 2).

**TABLE 2:** The [NiFe] H_2_-uptake hydrogenases of acidophilic verrucomicrobial methanotrophs.

**Organism**	**Oxygen tolerance**	**Uptake hydrogenase**	**Gene name^a^**	**Gene ID^b^**
		**group^a^**		
*Methylacidimicrobium cyclopophantes* 3B^c^	Sensitive	Group 1b	hynB^d^ hynA^e^	60379.peg.2444 60379.peg.2445
*Methylacidimicrobium fagopyrum* 3C	Sensitive	Group 1b	hynB^d^ hynA^e^	VER3v2_90073-4^f^ VER3v2_90072
*Methylacidimicrobium* strain LP2A	Sensitive	Group 1b	hynB^d^ hynA^e^	MAMLP_v1_11153 MAMLP_v1_11152
*Methylacidimicrobium tartarophylax* 4AC ^c^	Sensitive	Group 1b	hynB^d^ hynA^e^	60380.peg.187 60380.peg.186
*Methylacidimicrobium thermophilum* A8	Sensitive	Group 1b	hynB^d^ hynA^e^	MTHERMO_v1_1379 MTHERMO_v1_1378
*Methylacidiphilum fumariolicum* SolV	Tolerant^g^	Group 1d Group 1h	hyaB^d^ hyaA^e^ hhyL^d^ hhyS^e^	Mfumv2_1564 Mfumv2_1565 Mfumv2_0979 Mfumv2_0978
*Methylacidiphilum infernorum* V4	Tolerant	Group 1d	hyaB^d^ hyaA^e^	Minf_1320 Minf_1321
*Methylacidiphilum kamchatkensis* Kam1	Tolerant	Group 1d Group 1h	hyaB^d^ hyaA^e^ hhyL^d^ hhyS^e^	JQNX01_v1_10368 JQNX01_v1_10367 JQNX01_v1_60118 JQNX01_v1_60119
*Methylacidiphilum* sp. RTK17.1	Tolerant^g^	Group 1d	hyaB^d^ hyaA^e^	ANC58185.1 ANC58184.1

*^*a*^According to Søndergaard et al. (2016). ^*b*^Available at the MicroScope annotation platform. ^*c*^The draft genomes of strains 4AC and 3B are fragmented. ^*d*^Large subunit. ^*e*^Small subunit. ^*f*^The large subunit of the hydrogenase of strain 3C is present in two pieces. ^*g*^Conflicting data have emerged on the oxygen tolerance of the group 1d type of these strains.*

## Discussion

In this study, we have shown that the mesophilic *Methylacidimicrobium tartarophylax* strain 4AC is able to grow as an autotroph on hydrogen gas as sole energy source under oxygen-limited conditions. Hydrogen consumption by microorganisms is an ancient trait: H_2_ is thought to be the first energy source utilized by microorganisms on Earth (Lane et al., 2010). All known methanotrophs of the Verrucomicrobia phylum encode for one or more [NiFe] hydrogenases, but with distinct properties. These hydrogenases are very different in terms of oxygen tolerance, which could lead to niche differentiation of thermophilic and mesophilic methanotrophs in the natural geothermal environment.

The oxygen tolerance of strain 4AC for hydrogen consumption is low. Strain 4AC is able to grow autotrophically on hydrogen gas when the flux of oxygen is regulated to obtain a dO_2_ level in the cultivation system below the detection limit. The highest activity was observed when the oxygen concentration was limited to below 10 μM (8.1 O_2_ nmol ⋅ min^–1^ ⋅ mg DW^–1^). At oxygen-limited conditions, the maximum growth rate (μ_max_) on H_2_ (0.0048 h^–1^) is approximately 12% compared to the growth rate on methane (van Teeseling et al., 2014). The measured yield on H_2_ (2.1 ± 0.2 g DW ⋅ mol H_2_^–1^) is about 41% compared to that on CH_4_ (5.1 ± 0.2 g DW ⋅ mol CH_4_^–1^) and lower than the yield reported for *M. fumariolicum* strain SolV (3.4 g DW ⋅ mol H_2_^–1^), for ‘Knallgas’ bacteria like *Ralstonia eutropha* (4.6 g DW ⋅ mol H_2_^–1^; Morinaga et al., 1978) and for *Hydrogenomonas eutropha* (5 g DW ⋅ mol H_2_^–1^; Bongers, 1970). Considering the number of electrons available from CH_4_ (8e^–^) and H_2_ (2e^–^) assuming complete oxidation, the biomass increase is 0.043 and 0.085 mole CH_2_O (biomass) per electron for CH_4_ and H_2_, respectively, indicating that hydrogen might be a better electron source. The group 1b [NiFe] hydrogenases were thought to be restricted to strict anaerobes for the reduction of alternative electron acceptors (Greening et al., 2016). However, here we show that strain 4AC couples hydrogen oxidation to the reduction of molecular oxygen when the oxygen concentration is below 40 μM. Group 1b and 1d [NiFe] hydrogenases are more oxygen-sensitive and, theoretically, this could result in the net translocation of more protons per molecule of hydrogen oxidized compared to group 1h [NiFe] hydrogenases, which reflects their periplasmic localization (Cordero et al., 2019). We therefore provide strong evidence that group 1b [NiFe] hydrogenases are also involved in aerobic respiration.

The ability of strain 4AC to consume H_2_ is likely to be a universal trait shared among methanotrophic Verrucomicrobia. The H_2_-uptake [NiFe] hydrogenases of this guild can be divided over three distinct groups (Op den Camp et al., 2009; Sharp et al., 2014; van Teeseling et al., 2014). Genomic analyses revealed that all known mesophilic methanotrophic Verrucomicrobia encode a membrane-bound group 1b [NiFe] H_2_-uptake hydrogenase. In contrast, all their thermophilic counterparts encode a membrane-bound group 1d [NiFe] H_2_-uptake hydrogenase. In addition, the thermophilic strains, SolV and Kam1, possess a group 1h [NiFe] H_2_-uptake hydrogenase. In the betaproteobacterium *Ralstonia eutropha* H16, the group 1h type was found to be insensitive to oxygen, which is likely due to an unusual coordination of the proximal iron-sulfur cluster where a cysteine residue is replaced by an aspartic acid residue (Fritsch et al., 2011; Schäfer et al., 2016). In strain SolV, the group 1h [NiFe] hydrogenase oxidizes hydrogen under at least ambient oxygen, whereas autotrophic growth only occurs below an oxygen concentration of 1.5%. This difference could be explained by oxygen sensitivity of the group 3b [NiFe] hydrogenase, which likely couples H_2_ oxidation to CO_2_ fixation in *Methylacidiphilum* (Carere et al., 2017). However, this NADH-producing group 3b type is not necessarily needed for autotrophic growth on hydrogen gas, since it is absent in all mesophilic strains. As for the group 1d [NiFe] hydrogenase, this enzyme is only expressed and active under oxygen-limiting conditions in strain SolV (Mohammadi et al., 2017). However, inconsistent results have emerged from studies on the isolate *Methylacidiphilum* sp. RTK17.1, where the group 1d type appeared more tolerant toward oxygen (Carere et al., 2017). The answer for this oxygen tolerance could arise from the unusual coordination of the proximal iron-sulfur cluster, in which six instead of four cysteine residues are involved (Shomura et al., 2011). Apparently, other factors are at play that determine oxygen tolerance in the *Methylacidiphilum* strains. Altogether, the presence of only the group 1b [NiFe] hydrogenase in strain 4AC explains why growth only occurs under strong oxygen limitation, which likely applies to the other mesophilic strains as well.

Hydrogen consumption is a ubiquitous trait among phyla and indeed oxygen limitation largely determines the distribution of different hydrogenase types (Greening et al., 2016). Different types are found in a wide range of habitats, varying from the hypoxic hydrogen-rich animal guts to soils and waters with low H_2_ and high O_2_ availability. Group 1b [NiFe] hydrogenases are mostly found in hypoxic environments such as peat bogs (Greening et al., 2016). However, this hydrogenase type is also encoded by the human pathogen *Helicobacter pylori*, supporting aerobic H_2_ oxidation in microoxic environments (Olson and Maier, 2002; Greening et al., 2016). Indeed, group 1b [NiFe] hydrogenases were also detected in geothermal environments. The group 1b [NiFe] hydrogenases in the metagenomics survey of these geothermal environments, however, were encoded by members of the Aquificae phylum, and did not include *Methylacidimicrobium* members. We therefore propose that group 1b [NiFe] hydrogenases are more abundant in volcanic ecosystems than previously thought. Indeed, the mesophilic and thermophilic verrucomicrobial methanotrophs experience various oxygen concentrations in their natural geothermal habitat (Chiodini et al., 2001; Pol et al., 2007). The strong reduction potential of hydrogen may not only decrease the threshold for oxidizing methane (Hanczár et al., 2002), but H_2_ also sustains growth when methane is absent as was shown in this study and before in *Methylacidiphilum* (Carere et al., 2017; Mohammadi et al., 2017). In the natural ecosystem of strain 4AC, hydrogen levels in volcanic gasses are much higher than those of methane (Chiodini et al., 2001). Hydrogen gas might allow for the uptake of methane even at atmospheric concentrations, which was observed in the Solfatara ecosystem (Castaldi and Tedesco, 2005). The [NiFe] H_2_-uptake hydrogenases have been shown to be more widespread in the phylum Verrucomicrobia than previously thought (Greening et al., 2016), suggesting that Verrucomicrobia may play a role in the hydrogen cycle in different ecosystems.

## Conclusion

In conclusion, we show that strain 4AC can grow autotrophically on hydrogen gas but only under oxygen limited conditions using an oxygen-sensitive hydrogenase. This is the first study to show hydrogen oxidation by a mesophilic verrucomicrobial methanotroph. Apparently, the group 1b [NiFe] hydrogenase is not only functional in anaerobic respiration, but also in aerobic respiration at low oxygen. We propose that distribution of *Methylacidiphilum* and *Methylacidimicrobium* within acidic geothermal environments is influenced by the oxygen concentration, due to major differences in oxygen tolerance of the encoded hydrogenases. Therefore, we postulate that *Methylacidimicrobium* utilizes hydrogen gas and methane in acidic volcanic systems at moderate temperatures and low oxygen, similarly to the metabolism of its relatives of the *Methylacidiphilum* genus at higher temperatures and at various oxygen concentrations. This extends the evidence that verrucomicrobial methanotrophs are key players in consuming hydrogen and, therefore, these hydrogenases could aid in mitigation of greenhouse gasses.

## Data Availability Statement

The datasets generated for this study are available on request to the corresponding author.

## Author Contributions

SM, AP, MJ, and HC designed the project and experiments. SM and AP performed the experimental work. SM and AP maintained the chemostat cultures. TB performed the phylogenetic analysis. SM, RS, AP, and HC performed data analysis and data interpretation. RS, SM, and HC wrote the manuscript with feedback from the other authors. HC and MJ supervised the research.

## Conflict of Interest

The authors declare that the research was conducted in the absence of any commercial or financial relationships that could be construed as a potential conflict of interest.
