# Supplementary material for: The Acidophilic Methanotroph Methylacidimicrobium tartarophylax 4AC Grows as Autotroph on H2 Under Microoxic Conditions
Source: Front Microbiol. 2019 Oct 18;10:2352. doi: 10.3389/fmicb.2019.02352 (PMC6813726; doi:10.3389/fmicb.2019.02352)
Supplement: Supplementary file 1 [file Data_Sheet_1.PDF]

Supplementary Figure S1: Typical examples of exponential growth of strain 4AC in batch cultures on methane and hydrogen

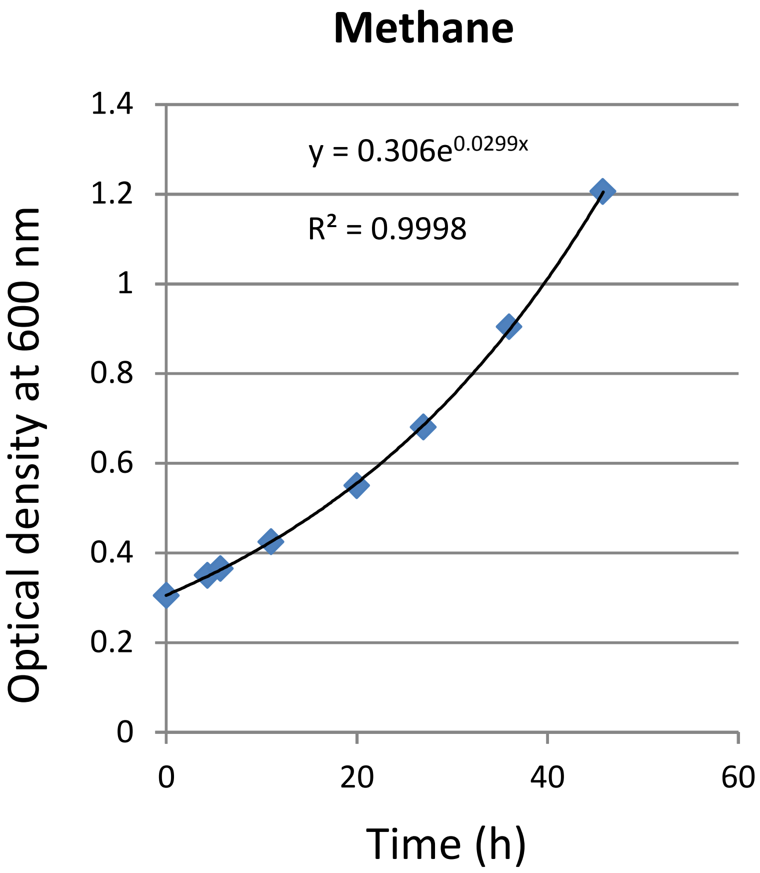

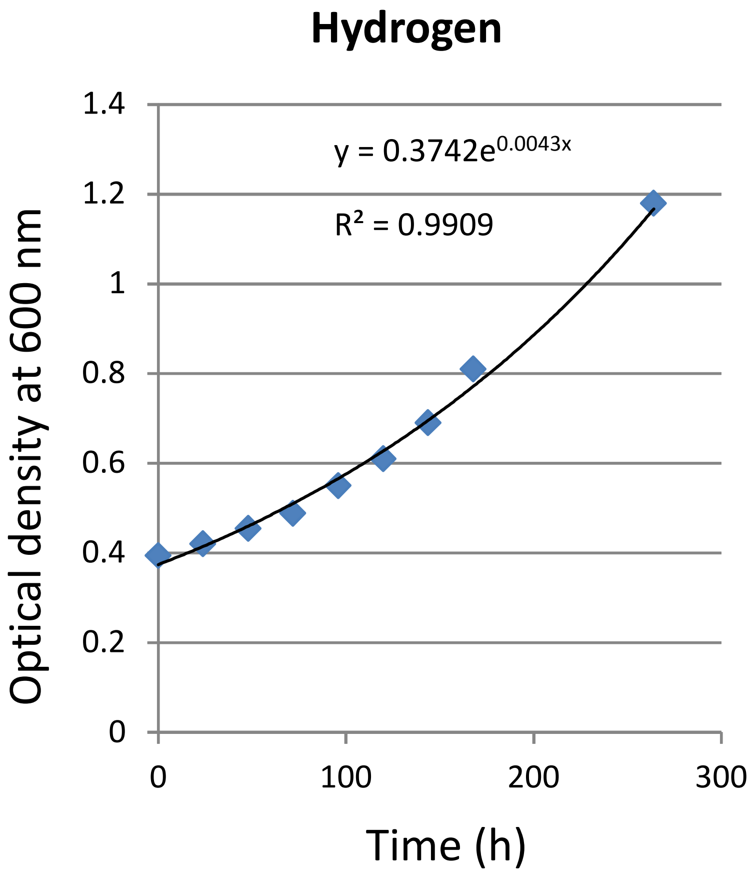

	Growth rate (h^{-1})	S.D. (n=3)
Hydrogen	0.0048	0.0006
Methane	0.033	0.003
